# Value of the loss of heterozygosity to *BRCA1* variant classification

**DOI:** 10.1038/s41523-021-00361-2

**Published:** 2022-01-17

**Authors:** Elizabeth Santana dos Santos, Amanda B. Spurdle, Dirce M. Carraro, Adrien Briaux, Melissa Southey, Giovana Torrezan, Ambre Petitalot, Raphael Leman, Philippe Lafitte, kConFab Investigators, Didier Meseure, Keltouma Driouch, Lucy Side, Carole Brewer, Sarah Beck, Athalie Melville, Alison Callaway, Françoise Revillion, Maria A. A. Koike Folgueira, Michael T. Parsons, Heather Thorne, Anne Vincent-Salomon, Dominique Stoppa-Lyonnet, Ivan Bieche, Sandrine M. Caputo, Etienne Rouleau

**Affiliations:** 1 Department of Genetics, Institut Curie, 26 rue d’Ulm, Paris, France; 2 Department of Medical Biology and Pathology, Gustave Roussy, Cancer Genetics Laboratory, Gustave Roussy, Villejuif, France; 3 Department of Clinical Oncology, A.C. Camargo Cancer Center, São Paulo, Brazil; 4 Department of Oncology, Center for Translational Oncology, Cancer Institute of the State of São Paulo - ICESP, São Paulo, Brazil; 5 Centro de Oncologia Hospital Sírio Libanês-São Paulo, São Paulo, Brazil; 6 Department of Genetics and Computational Biology, QIMR Berghofer Medical Research Institute, Brisbane, QLD Australia; 7 Molecular Biology and Genomics Group, A.C. Camargo Cancer Center, São Paulo, Brazil; 8 National Institute of Science and Technology in Oncogenomics and Therapeutic Innovation, PSL Research University, Sao Paulo, Brazil; 9 Research University, PSL, Paris, France; 10 Laboratory of Clinical Biology and Oncology, Centre François Baclesse, Inserm U1245 Genomics and Personalized Medicine in Cancer and Neurological Disorders, Normandy University, Caen, France; 11 Department of Pathology, The University of Melbourne, 3010 Victoria, Australia; 12 Department of Pathology, Institut Curie, 26 rue d’Ulm, Paris, France; 13 Wessex Clinical Genetics Service, University Hospital Southampton NHS Foundation Trust, Southampton, UK; 14 Wessex Regional Genetics Laboratory, Salisbury NHS Foundation Trust, Salisbury, UK; 15 Laboratory of Human Molecular Oncology - Centre Oscar Lambret, Lille Cedex, France; 16 Inserm U830, University Paris Descartes, Paris, France; 17 Faculty of Pharmaceutical and Biological Sciences, University of Paris, F-75006 Paris, France

**Keywords:** Cancer genetics, Cancer genomics

## Abstract

At least 10% of the *BRCA1/2* tests identify variants of uncertain significance (VUS) while the distinction between pathogenic variants (PV) and benign variants (BV) remains particularly challenging. As a typical tumor suppressor gene, the inactivation of the second wild-type (WT) *BRCA1* allele is expected to trigger cancer initiation. Loss of heterozygosity (LOH) of the WT allele is the most frequent mechanism for the *BRCA1* biallelic inactivation. To evaluate if LOH can be an effective predictor of *BRCA1* variant pathogenicity, we carried out LOH analysis on DNA extracted from 90 breast and seven ovary tumors diagnosed in 27 benign and 55 pathogenic variant carriers. Further analyses were conducted in tumors with PVs yet without loss of the WT allele: *BRCA1* promoter hypermethylation, next-generation sequencing (NGS) of *BRCA1/2*, and BRCAness score. Ninety-seven tumor samples were analyzed from 26 different *BRCA1* variants. A relatively stable pattern of LOH (65.4%) of WT allele for PV tumors was observed, while the allelic balance (63%) or loss of variant allele (15%) was generally seen for carriers of BV. LOH data is a useful complementary argument for *BRCA1* variant classification.

## Introduction

Monoallelic germline *BRCA1/2* pathogenic variants (PV) substantially increase the risk of developing breast (BC) and/or ovarian cancer (OC), but at least 10% of *BRCA1/2* tests result in a variant of unclassified/uncertain/unknown significance (VUS)^1^. The distinction between germline pathogenic and benign nature of a missense variant remains particularly problematic, while the classification of a rare germline missense variant remains challenging^2^. In addition to genetic counseling implications, *BRCA1/2* variant classification now has an important impact on therapeutic decisions as well as predicting the benefit from PARPi^3,4^ and DNA-damaging agents^5^. Recent data suggest that in addition to the germline PV, locus-specific LOH may also be necessary to predict sensitivity to DNA-damaging agents for better outcomes^6–8^. Several tests assessing different patterns of LOH have also been prospectively evaluated in clinical trials to infer the response to PARPi^9–11^. The estimations of the second event have been biased, as most of the LOH reported have copy-neutral allelic imbalance^12^.

A recent report of LOH analysis in *BRCA1/2* locus of 160 tumors with germline PV (94% patients with truncating variants) confirmed a proportion of loss of wild-type (WT) allele in ovarian tumors as high as 93% for *BRCA1* and 90% for *BRCA2* carriers^6^. A similar percentage of 90% occurred for *BRCA1* breast cancers but was less evident (54%) for *BRCA2* breast cancers^6,13^. In contrast, for sporadic tumors without any mutated allele, the LOH of the 17p is a more common event than a focal deletion around *BRCA1*. This is found in 20–50% of sporadic breast cancers^14^ and up to 87% of ovarian cancers^15^.

Many approaches have been proposed to assist the classification of *BRCA1/2* germline missense VUS, including analysis of splicing effects^16^, co-segregation studies within families^17,18^, co-occurrence in *trans* with a PV, personal*/*family history, and histopathologic profile^18,19^. Currently, LOH data is not included in multifactorial likelihood model calculations. Although some studies have argued in favor of LOH as a useful tool to predict variant pathogenicity^20–22^, others warned that it should be applied with caution^12,23,24^. Part of the disagreement may be explained by the difference in the methodology used for the analysis. Initially, the presence of LOH was performed using fragment analysis of microsatellite repetition to evaluate if both alleles were present. The size of the *BRCA1* locus could be a hurdle for this evaluation. Nowadays, LOH analysis is performed with more sensitive and precise methods, such as next-generation sequencing or pyrosequencing, which are also able to take into account intratumor heterogeneity. Furthermore, since the probability of the presence of LOH of the WT allele by chance is not null, it seems important to explore the LOH status on several tumors with the same germline variant^25^.

We tested the hypothesis that the inactivation of the WT allele at the tumor level could argue in favor of *BRCA1* variant pathogenicity. For this purpose, we evaluated 97 tumor samples (90 breast and seven ovarian) from carriers of 26 distinct *BRCA1* germline variants (ten PV, eight VUS, and eight BV) using a pipeline (pyrosequencing, NGS, methylation, and BRCAness analysis) to identify genomic markers of *BRCA1* locus-specific LOH and other possible mechanisms of gene inactivation.

## Results

### Clinical, pathological, and genetic data

We examined 97 breast/ovarian tumor samples from a total of 94 patients. All seven ovarian tumors were high-grade serous ovarian carcinomas. The majority of samples corresponded to breast carcinomas (*n* = 90; 93%), but with one ductal carcinoma in situ. The 90 breast tumors were mostly ductal (90%), high-grade (70%), and estrogen⁄progesterone-receptor-negative breast cancers (~60%; Table 1).

**Table 1:** Number of breast and ovarian samples analyzed for each variant category. Clinical and pathological data of the breast carcinoma cohort.

	Pathogenic	(likely) Benign	VUS^*^	Total
Ovary	4	1	2	7
Breast	51	26	13	90
**BREAST**				
Invasive	51	25	13	89 (99%)
In situ	0	1	0	1 (1%)
**Type**				
Ductal carcinoma	48	22	11	81 (90%)
Other types	3	4	2	9 (10%)
**Grade**				
Grade 1	1	5	0	6 (7%)
Grade 2	4	10	2	16 (17%)
Grade 3	45	10	9	64 (70%)
Unknown	1	1	2	4 (4%)
**Estrogen receptor**				
Positive	7	21	5	33 (36%)
Negative	39	4	8	51 (57%)
Unknown	5	1	0	6 (6%)
**Progesterone receptor**				
Positive	5	16	3	24 (26%)
Negative	39	6	9	54 (61%)
Unknown	7	4	1	12 (13%)
**Her2 status**				
Positive	4	1	1	6 (7%)
Negative	26	21	10	57 (64%)
Unknown	21	4	2	27 (29%)
**Triple-negative**^§^				
TN	23	3	6	32 (36%)
Other subtypes or unknown	28	23	7	58 (64%)
**Age**				
<50 years	20	16	6	42 (47%
≥50 years	4	9	5	18 (20%)
Unknown	27	1	2	30 (33%)

^*^Variant of uncertain clinical significance.

^§^In this part, only triple-negative breast cancers for which we had complete information about ER, PR, and Her2 status are presented.

**Fig. 1:**
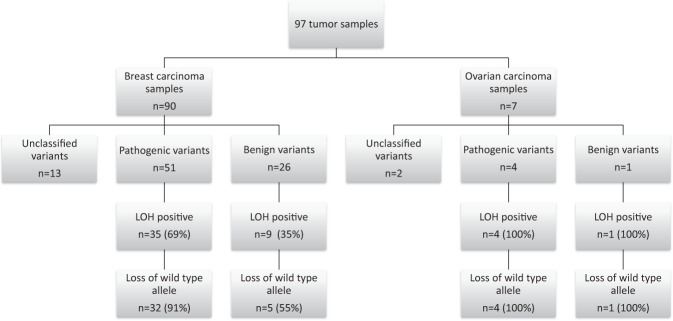
Summary of *BRCA* locus-specific LOH status of breast and ovarian tumors from individuals with germline *BRCA1* variants. In this figure are presented all the results obtained according to the type of tumor and the class of variant identified.

These 94 patients carried 26 different *BRCA1* variants: ten pathogenic, eight (likely) benign, and eight VUS (Supplementary Data 1 and Fig. 1). Among the PV, there were four missenses and six nonsense/frameshift (one skipping of exon 23 and one large duplication). As shown in Table 1, 76% of the breast tumors from PV carriers were estrogen and progesterone-receptor-negative. The tumors were mainly grade 3 (45/51, 88%) and diagnosed before age 50 years. In the 65 cases whose Her2 status was assessed, 6 (9%) were Her2 positive breast carcinomas, of which four were from PV carriers (two over 50 years, two with an onset of unknown age)^26^.

### Somatic loss of WT allele correlates with the pathogenic classification of *BRCA1* germline variants

An analysis pipeline was established for the identification of genomic markers for *BRCA1* locus-specific LOH by pyrosequencing or NGS. Pyrosequencing was the method applied to detect any allelic imbalance of the variant for tumor samples. Assuming that gene inactivation of both alleles is expected for a pathogenic variant in a tumor suppressor gene, observation of an allelic balance identified for a pathogenic variant was subject to three additional assays in order to further explore the mechanism of tumorigenesis and second allele inactivation: (1) *BRCA1* promoter hypermethylation analysis, (2) Next-generation sequencing (NGS) of *BRCA1* for an inactivating variant at the somatic level, and (3) BRCAness signature analysis.

**Fig. 2:**
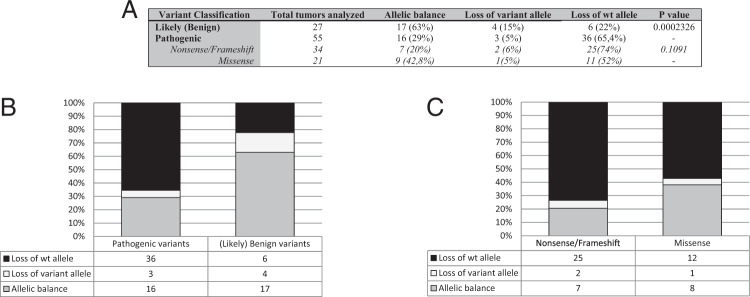
Proportion of breast/ovarian samples presenting loss of wt allele among pathogenic and (likely) benign variants considering variant effect at the protein level. **A** Results obtained according to the class of the variant. For the pathogenic variants, there are also results according to the type of variant: missense or Nonsense/Frameshift; **B** Histogram of the results according to the class; **C** Histogram representation of the results for the pathogenic variants according to the type of variant.

Considering the entire cohort (Fig. 2), germline PV presented LOH in 70% of tumors, of which 65.4% presented a LOH of the wild-type allele. Loss of WT of a germline *BRCA1* variant in the tumor predicts pathogenicity with a sensitivity of 65% and specificity of 78%. The percentage of samples associated with LOH of the wild-type allele was different according to the nature of the variant. Frameshift variants were more likely to present loss of wild-type allele than missense variants (74% vs 52%, not statistically significant) (Fig. 2).

Germline BVs presented with LOH in 37% of the breast and ovarian tumors combined, and only 22% of these were due to loss of the wild-type allele (Fig. 2).

In regard to breast cancer samples, LOH was observed in 69% (35) of tumors with a germline PV (51), within which the loss of the wild-type allele was present in 91% (32 of 35) while the overall allelic balance was observed in 31% (16) carrying PVs (Table 2). In contrast, LOH was observed in 35% of tumors with germline BVs, and among the nine samples that presented LOH, 56% (5) were due to the loss of the wild-type allele (Fig. 1 and Table 2).

**Table 2:** LOH breast/ovarian cancer results from pyrosequencing or NGS experiments for pathogenic (P), benign (B), and likely benign (LB) variants.

Variant nomenclature	Protein nomenclature	Impact	Variant class	Allelic balance	Loss of variant allele	Loss of wt allele	Total	% of allelic imbalance/LOH	% LOH wt
**BREAST**									
c.68_69del	p.Glu23Valfs*17	FS	P	2	0	7	9	78%	100%
c.131G>T	p.Cys44Phe	MS	P	2	0	1	3	33%	100%
c.181T>G	p.Cys61Gly	MS	P	5	0	6	11	55%	100%
c.962G>A	p.Trp321Ter	NS	P	0	1	1	2	100%	50%
dupEx3-8 (c.81_547dup)	p.Gly183Valfs*4	FS	P	1	0	1	2	50%	100%
c.5095C>T	p.Arg1699Trp	MS	P	1	0	2	3	67%	100%
c.5123C>A	p.Ala1708Glu	MS	P	0	1	0	1	100%	–
c.5266dup	p.Gln1756Profs*74	FS	P	3	1	13	17	84%	87%
c.5324T>G	p.Met1775Arg	MS	P	1	0	1	2	50%	50%
c.5453A>G	splicing exon 23 (p.(Gly1803Glnfs*11))	FS	P	1	0	0	1	–	–
**Total pathogenic**	–	–	P	16	3	32	51	69%	91%
c.1067A>G	p.Gln356Arg	MS	B	4	2	1	7	43%	33%
c.2477C>A	p.Thr826Lys	MS	B	1	1	0	2	50%	–
c.4535G>T	p.Ser1512Ile	MS	B	2	1	2	5	60%	66%
c.4812A>G	p.Gln1604Gln	SYN	LB	2	0	1	3	33%	100%
c.4955T>C	p.Met1652Thr	MS	LB	1	0	0	1	–	–
c.4956G>A	p.Met1652Thr	MS	B	5	0	0	5	–	–
c.5117G>C	p.Gly1706Ala	MS	B	1	0	1	2	50%	100%
c.5531T>C	p.Leu1844Pro	MS	B	1	0	0	1	–	–
**Total (likely) benign**	–	–	B/LB	17	4	5	26	35%	56%
**OVARIAN**									
c.181T>G	p.Cys61Gly	MS	P	0	0	1	1	100%	100%
c.2477C>A	p.Thr826Lys	MS	B	0	0	1	1	100%	100%
dupEx3-8 (c.81_547dup)	p.Gly183Valfs*4	FS	P	0	0	1	1	100%	100%
c.5266dup	p.Gln1756Profs*74	FS	P	0	0	1	1	100%	100%
c.5324T>G	p.Met1775Arg	MS	P	0	0	1	1	100%	100%

*MS* missense, *NS* nonsense, *SYN* synonymous, FS: Frameshift.

Noteworthy that loss of WT allele was present in all seven ovarian cancer samples (four PV, two VUS, and one BV carriers; Fig. 1, Table 2, and Supplementary Table 5).

### Alternative second hit event for PVs without loss of the WT allele

Allelic balance was observed in tumors from 15 PV carriers (Fig. 2). Complete coding sequencing of *BRCA1/2* in tumor DNA by NGS did not reveal any further *BRCA1* somatic PV. One sample with loss of the variant allele was observed with *BRCA1* promoter methylation (Supplementary Table 4).

### Correlation between LOH presence and high genomic instability score

The analysis of genomic instability was performed for the establishment of BRCAness score in a set of 12 available frozen samples: six PV, three BV, and three VUS (Supplementary Table 5). BRCAness score showed a strong correlation with LOH analysis.

Of the six PVs, four with loss of wild-type allele had a high BRCAness score and two with allelic balance had a low BRCAness score. Of the 3 BVs, all showed allelic balance and low BRCAness score (all luminal BCs).

### LOH analysis of tumors from VUS carriers

Fifteen tumor samples from germline missense VUS (eight unique variants) were available for analysis (Tables 3 and 4). Several tumors with the same variant were available for only two variants, both located in the BRCT domain: c.5497G>A (three samples) and c.4963T>C (five samples). All three samples carrying the variant c.5497G>A presented loss of WT allele. Four out of five samples with c.4963T>C variant presented loss of the wild-type allele. To confirm our hypothesis, we performed two functional assays to evaluate the impact of the variant c.4963T>C: (i) evaluation of the effectiveness of double-strand breaks repair by HR, (ii) production and purification of mutated BRCT domain (Supplementary Figs. 1, 2)^27^. The team of Dr. Caputo has already performed these functional tests for the *BRCA1* c.5497G>A variant previously^27^. In the presence of the c.4963T>C variant, DNA repair by HR was deficient and the BRCT domain was poorly soluble so it could not be purified. We also had enough co-segregation data to establish causality scores using the multifactorial model. The results obtained were consistent with pathology and functional data which allowed us to classify both c.4963T>C and c.5497G>A variants as PVs (Tables 5–7 and Supplementary Fig. 4).

**Table 3:** LOH results for tumor samples from a variant of uncertain significance (VUS) carriers.

Variant	Protein	Variant class	Impact	Type of tumor	LR pathology	Allelic balance	Loss of wt allele	Loss of variant allele	Total	% of allelic Imbalance/LOH	% LOH wt
c.3074C>T	p.Thr1025Ile	VUS	MS	Breast	4.41	1	0	0	1	–	–
c.4841C>T	p.Pro1614Leu	VUS	MS	2 Breasts		0	1	1	2	100%	50%
c.4963T>C	p.Ser1655Pro	VUS	MS	4 Breasts, 1 Ovary	152.88	1	4	0	5	80%	100%
c.5057A>G	p.His1686Arg	VUS	MS	Breast	3.73	0	1	0	1	100%	100%
c.5072C>A	p.Thr1691Lys	VUS	MS	Breast	4.41	1	0	0	1	–	–
c.5177G>T	p.Arg1726Ile	VUS	MS	Breast	0.21	1	0	0	1	–	–
c.5203G>A	p.Glu1735Lys	VUS	MS	Breast	0.64	0	1	0	1	100%	100%
c.5497G>A	p.Val1833Met	VUS	MS	2 Breasts, 1 Ovary		0	3	0	3	100%	100%

**Table 4:** Available evidence about the VUS analyzed in this study MS=missense; C-E: Evidence of pathogenicity of the unclassified variant *BRCA1* c.4963T>C.

Variant	Protein nomenclature	Functional domain	dbSNP	Frequency gnomAD (V2.1.1)	SIFT	Align GVGD^*^	CADD	References
c.3074C>T	p.Thr1025Ile	–	rs397509034	–	0.26	C0	18.61	–
c.4841C>T	p.Pro1614Leu	–	rs766305255	ALL:0.0012% - NFE:0.0027%	**0.03**	C0	11	
c.4963T>C	p.Ser1655Pro	BRCT1	rs1057518639	–	**0.01**	C0	24.8	^39^
c.5057A>G	p.His1686Arg	BRCT1	rs730882166	–	**0**	C25	26.3	^28,37^
c.5072C>A	p.Thr1691Lys	BRCT1	rs80357034	–	**0**	C65	25.4	^28,37,40^
c.5177G>T	p.Arg1726Ile	BRCT1	rs786203547	–	0.07	C0	22.8	^28^
c.5203G>A	p.Glu1735Lys	BRCT1	rs397509238		**0**	C55	26.3	^28^
c.5497G>A	p.Val1833Met	BRCT1	rs80357268	ALL:0.00041% - NFE:0.00090%	**0.01**	C0	33	^28,37,38^

^*^Vallée et al., Huam Mutation, 2016.

For SIFT, bold = deleterious by SIFT.

**Table 5:** Loss of heterozygosity analysis of breast and ovarian tumors of family 1 carriers.

Patient	Tumor	% of viable tumor cells	Tumor histology	VAF tumor	LOH
1	1	60%	Triple-negative breast invasive carcinoma of no special type	65%	Yes
	2	30%	Triple-negative breast invasive carcinoma of no special type	49%	No
2	1	70%	Triple-negative breast invasive carcinoma of no special type	88%	Yes
	2	90%	Ovarian high-grade serous carcinoma	67%	Yes
3	1	90%	Positive lymph node from luminal breast cancer (ER/PR positive, HER2 negative)	70%	Yes

*VAF* variant allele frequency.

**Table 6:** Clinical, pathological and co-segregation data available for the variant *BRCA1* c.4963T>C (Suppl. Fig. 4 presents the pedigree of F1 family).

Family	Origin	Index case history of cancer	Family history of cancer	LR pathology	LR Co-segregation
F1	Brazil	TNBC (29 y and 45)	Sister (Luminal BC 29 y) [+]; father (hepatocarcinoma at 59 y) [+]; one paternal aunt (HGSOC 45 y and TNBC 60 y) [+]; two paternal aunts with breast cancer (49 y [+] and 70 y); one paternal uncle with prostate cancer; one paternal uncle (kidney cancer at 32 y); one paternal uncle (leukemia at 45 y); one paternal uncle (hepatocarcinoma at 80 y); paternal grandmother (pancreatic cancer at 47 y); paternal grandfather (hepatocarcinoma at 60 y)	6.57972	4.608
F2	United Kingdom	TNBC (47 y)	Paternal aunt (ovarian ca 49 y) [+], two paternal aunts (Breast ca 50 y), paternal cousin (Bilateral breast ca 29 y and 37 y) [+]	13.9129	7.54899
F3	United Kingdom	Breast ca (37 y)	Maternal and paternal history of breast cancer		NA
F4	United Kingdom	Breast ca (31 y)	Not available	1.67	NA
F5	United Kingdom	HGSOC	Not available		NA
F6	United Kingdom	Ovarian ca (48 y)	Mother (ovarian ca at 45 y) [+]; maternal grandmother (ovarian ca 71 y); maternal uncle (prostate ca 55 y)		1.96755

*TNBC* triple-negative breast cancers, *HGSOC* high-grade serous ovarian carcinoma, *BC* breast cancer, *ca* cancer, [+] carrier of variant, [−] non-carrier of variant.

**Table 7:** Classification of the *BRCA1* VUS c.4963T>C and c.5497G>A based on multifactorial score.

Variant	Prior probability	LR co-segregation	LR pathology	Family history	Odds for causality	Posterior probability of pathogenicity	Class
c.4963T>C, p.Ser1655Pro	0.03	68.44	152.88	8.71	91,176.32	0.9996	5
c.5497G>A, p.Val1833Met	0.03	7.85814	80.4827	2.76	442.75	0.9986	5

For the BRCT domain VUS c.4841C>T, two samples were available but with discordant results. One showed loss of variant while the other loss of WT allele.

For five VUS, only one tumor was available. Three VUS presented allelic balance: c.3074C>T, c.5072C>A, and c.5177G>T. The two remaining BRCT VUS showed loss of the WT allele (c.5057A > G and c.5203G>A). The functional assays performed in this study evaluated only the variants located in the BRCTs domains. All other variants of the BRCTs domains had been studied by Petitalot et al.^27^.

For VUS carriers with frozen breast tissue samples available (c.4841C>T and c.3074C>T), analysis of genomic instability was also performed. All three tumors (one luminal and two triple-negative breast carcinomas) showed a low BRCAness score (Supplementary Table 5).

### Simulation of the minimum number of LOH status for classification

A simulation study was used to estimate the number of cases needed to predict the variant classification (Supplementary Tables 2, 3). The main question that remained was related to the minimum number of samples with LOH in relation to the number of samples analyzed to classify a variant. Thus, for LOH information to be applicable, a simulation study was proposed to estimate the minimum number of samples and of the LOH results to classify a variant (Supplementary Tables 2, 3). For this, we considered the hypothesis that the variable loss of WT follows the binomial distribution law and compare the distribution of BV and PV.

The threshold was determined based on Plon et al. classification^28^. The accepted risk for class 2 was below 0.05%. The main goal here was then to exclude the neutrality if there were enough samples with LOH. One or two samples with LOH were insufficient since the probability to have LOH was still high for a BV. With five out of five samples presenting LOH (Supplementary Tables 2), the probability to have this situation with a BV was below 0.05%. That is the first situation, which can lead to a decision. Therefore, one can consider the minimum number of analyzed samples to predict variant classification should be at least five samples. On the other hand, if there is not enough LOH, one can exclude pathogenicity. The probability for a PV to present one out of five samples with LOH is below 1% (Supplementary Table 3). Supplementary Tables 2–3 and 6–8 can be used to weigh the result on LOH and decide whether or not to take into account this information.

For variants with at least three samples, access was available in our cohort for two VUS, with loss of the WT allele present in 80% (4/5) for c.4963T>C and 100% (3/3) for c.5497G>A. Thus, for those two variants, LOH analysis can moderately exclude neutrality but additional arguments are still required to conclude pathogenicity. In those cases, the probability for a BV is close to 1% (Supplementary Table 2). The remaining two BRCT VUS showed loss of the WT allele (c.5057A>G and c.5203G>A) in only one tumor. This situation is rare but possible for BV (22%). Therefore, we cannot classify them solely based on this data.

## Discussion

The loss of the remaining WT allele is the last event before entering germline *BRCA1*-related tumorigenesis. This mainly happens through locus-specific LOH of the WT allele^29^. LOH in sporadic breast and ovarian cancer is not rare, but the lost allele is random. We hypothesized that the repetitive observation of the loss of the WT allele for the same variant should argue in favor of the variant pathogenicity. Thus, we compared pretreatment tumor biopsies of pathogenic (55 samples) and (likely) BV carriers (27 samples). Combining NGS and pyrosequencing, a consistent pattern of predominance in PVs showed 69% allelic imbalance with 91% loss of wild-type for PVs, while (likely) BVs showed 35% allelic imbalance with 55% loss of WT allele, which was consistent with different previous reports^6,25,30^.

Several studies confirmed that most tumors with germline *BRCA1* PVs have locus-specific LOH^12,22,24^. We searched for other inactivation mechanisms in tumors with PVs yet without loss of the WT allele (Supplementary Table 4). No further *BRCA1/2* somatic inactivating variant was identified in tumor samples by NGS. Promoter hypermethylation was identified in one BC sample and a PIK3CA mutation in five BC samples (Supplementary Table 4). Two presented luminal phenotypes, not typically related to *BRCA1* carcinogenesis, while histopathology data were not available for the remaining three. We searched for PIK3CA hot spot mutations in parallel to confirm tumoral cellularity, since it is rarely detected in *BRCA1*-related TNBC^31^. The remaining cases may be explained by stromal contamination related to low tumor cellularity (less than 30% in 2 samples), DNA sample quality, and limitations of pyrosequencing to identify the allelic imbalance.

Nevertheless, the application of this methodology could raise some limitations. Some issues could mask the results, such as non-tumor tissue contamination, low tumor cellularity, low quality of tumor DNA, and tumor heterogeneity. Thus, performing macro-dissection to separate tumor tissue from healthy breast tissue was an important step for the identification of LOH when present. Tumor cell heterogeneity as well as heterogeneity in the mechanism of WT allele inactivation may also exist within the tumor. For one sample carrying the variant c.4535G>T, different patterns of LOH were observed when these different regions were analyzed separately. This observation has already been highlighted in literature with sporadic breast cancer in a germline PV carrier^26^. It is also consistent with previous data of Klaes C. et al., concluding that different mechanisms inactivating the wild-type allele may be present within the same tumor at various extents^12^. This heterogeneity and technical limitations could also be a challenge to assess the correct status for LOH and explain the need to analyze multiple cases with the same variant. For repeated analysis, the rarity of the variants and the difficulty in grouping families with several tumors carrying the same sequence variation can also be a limitation for this analysis where a minimum number of samples were necessary to reach a conclusion. However, instead of co-segregation studies, this approach could be performed using stored samples from individuals with multiple primary tumors and from families with many affected individuals.

We further evaluated the occurrence of LOH according to the effect of the variant on protein level. LOH was reported for 52 and 74% of pathogenic missense and frameshift/nonsense variants, respectively. Although this difference was not statistically significant, this observation could be in favor of some dominant-negative effect of *BRCA1* missense PVs. Vaclova et al. showed that lymphoblastoid cell line of heterozygous BRCT missense variants carriers presented a lower level of BRCA1 recruitment into DNA-damaged foci and a higher sensitivity to PARPi than cells with truncating variants or normal cells, suggesting that the intact protein is unable to function normally in the presence of mutant *BRCA1*^32^. This trend has also been shown for other DNA repair proteins such as ATM, POLE1, and TP53^33–35^. In fact, there is increasing evidence that heterozygous *BRCA1* PVs lead to haploinsufficiency of some BRCA1 functions, even for the homologous recombination activity that happens before the loss of heterozygosis^36^. In the current study, the rate of LOH of the WT allele for pathogenic variants is indeed a bit lower (65,4%) than other reports^6,37^. However, in the work of Maxwell et al. half of the samples of breast and ovarian tumors came from population studies from TCGA and included only 10% of samples containing missense variants. An LOH rate of 90 and 93% in breast and ovarian tumors, respectively, was described. In the most recent study of Hauke et al., only ovarian cancer samples were analyzed, and a minority (less than 10%) contained missense variants with a LOH of WT rate of 88% (Supplementary Fig. 5).

Regarding the use of the loss of heterozygosity for *BRCA1* variant classification, there are discordant conclusions^12,23^. Such dissemblance is mainly related to the different characteristics of the studied population as for example the predominant tumor type (breast *vs.* ovary), the predominant variant type (missense vs frameshift) in the sample, as well as the differences in the sensitivity of the methodology used for LOH analysis. We hypothesized that the repetition of LOH of the wild-type for tumors from carriers of the same variant could help with the classification of the VUS. Using a more sensitive approach based on NGS and pyrosequencing, we observed a difference in LOH patterns for known pathogenic and (likely) BVs. This result, if validated with a much larger sample set, would indicate that the LOH pattern is seen by NGS. It may also provide additional information for the classification of VUS in *BRCA1* if the LOH is observed in several cases with the same variant. Indeed, considering the sensitivity and specificity of the LOH analysis seen in this study, we reinforce that the analysis of only one case does not allow the classification of the variant in question. LOH information may be complementary to histopathological features, helping to refine the cases with a low pathology likelihood ratio (LR). LOH status was compared with LR pathology score, currently incorporated into the multifactorial model for VUS classification (Supplementary Fig. 3). In most cases, both analyzes are consistent. However, a loss of the WT allele was observed despite the low LR pathology for three VUS. To confirm this hypothesis, multivariate analysis of LOH and pathology data in a larger number of samples is required. Our results also confirm that the information on a unique case should not be used by any means as an argument for VUS reclassification. To reduce the risk of a misleading conclusion, the number of tumor samples should be at least three to exclude neutrality at 1% (if all present LOH) and better at five samples to exclude neutrality at 0.05% (if all present LOH). The estimated probability seen in Supplementary Tables 2 and 3 can be useful in deciding whether to use LOH results for the exclusion of variant neutrality or pathogenicity. However, the approach proposed in these tables does not aim to be a statistical test, but rather a simulation of the distribution of the number of samples with an LOH according to the variant classification (benign or pathogenic). Therefore, this approach is an ad hoc heuristic for further workup on a variant. By itself, this approach is not able to classify a variant, but it can help to prioritize the collection of evidence with tumoral samples. In fact, in our specific case (the *BRCA1* gene and breast cancer), the LOH of the wild-type allele is not systematic, but the probability is sufficient to propose the likelihood of neutrality or pathogenicity. Our approach can adapt the model to different types of cancers or other genes. For breast cancer, we can propose to add a different weight in the LR pathology score according to LOH recurrency.

We were able to support our hypothesis concerning LOH by carrying out functional assays and then using the multifactorial model for two variants (c.5497G>A and c.4963T>C). Up until then, both variants remained unclassified based on multifactorial analysis because of insufficient data to establish a causality score, but most data favored causality without consensus^27,38,39^. For the variant c.5497G>A, all three samples presented loss of the WT allele. For the variant c.4963T>C (also in BRCT domain), four out of five samples (four breast and one ovarian cancer) presented loss of the WT allele, and the only sample presenting allelic balance had low tumor cellularity (30%). The binomial distribution of the probability of LOH in BV helped to exclude the neutrality with a probability of error under 1% (Table 2 of supplementary data). Functional assays argued in favor of pathogenicity for both variants^38^. Here, we could demonstrate a functional impact of the variant c.4963T>C through the destabilization of the BRCT domain and in HR performance, which would be arguments in favor of its pathogenicity. The study of c.5497G>A had been carried out previously^27^. Further, we were able to gather additional data which allowed us to establish a causality score sufficient to classify these variants as pathogenic (Table 7).

Finally, the absence of LOH of the WT allele could be an argument against platinum salts or PARPi treatment. Currently, *BRCA1/2* PVs are considered biomarkers of response to platinum salts and PARPi without considering the LOH status^5,11^. Recent data on ovarian cancer suggest that the finding of a PV is not enough to predict primary resistance for these agents and confirms that LOH analysis at the tumor level and the presence of BRCAness phenotype may refine this prescription by identifying those patients who will respond positively^5,6^. We identified loss of the variant allele in 3 (5%) of PV carriers and allelic balance in 16 (31%) breast tumor samples. In the set of PVs, 19 of 51 tumors analyzed lacked locus-specific LOH and showed low genomic measures of BRCAness. Thus, the absence of inactivation of the WT allele can be a potential risk for primary resistance. Also, the presence of LOH in breast cancer should help to identify the subset of patients with the greatest benefit from PARPi^25^.

In conclusion, these results emphasize that tumors associated with *BRCA1* germline variants should not be considered uniformly from a tissue, pathologic, morphological, and genetic point of view. We propose to incorporate LOH data for variant pathogenicity prediction since tumoral sequencing, LOH information, and HRD score is increasingly available with PARPi indications. Besides being a complementary argument to help in the classification of *BRCA1* variants, LOH could be used as an additional biomarker of response to PARPi even with *BRCA1* PVs.

## Methods

### Ethics declaration

All patients recruited at A.C. Camargo provided written informed consent and the study was approved by the Internal Ethics Committee Board (#1754/13). Informed written consent was also provided by all participants of the kConFab Consortium and the study was approved by the Peter MacCallum Cancer Centre Human Research Ethics Committee (#97/27). All patients recruited at Institut Curie provided written informed consent and the study was approved by the Internal Ethics Committee Board.

### Patients and tumor/DNA samples

The patients were all index cases from high-risk breast and/or ovarian cancer from French, Australian, and Brazilian families with eligibility criteria for screening of *BRCA1/2* PV according to local/national consensus statement who had consented for genetic testing and use of their samples for research studies.

Paraffin-embedded tumor pretreatment biopsies from 90 breast cancer patients and seven ovarian cancer patients carrying 26 distinct *BRCA1* variants were obtained from the kConFab consortium (*n* = 29), French biological resource centers of Institut Curie (*n* = 62), Centre Oscar Lambret (*n* = 1), and A.C. Camargo Cancer Center (*n* = 5). Slides of each tumor specimen, stained with hematoxylin and eosin, were reviewed by a local pathologist, who then performed macro-dissection to separate tumor epithelium from the surrounding stroma and healthy tissue and estimated the percentage of tumor cellularity. Tumor DNA extraction from 6–10 μm-sections of formalin-fixed paraffin-embedded (FFPE) tissues was performed using a NucleoSpin 8⁄96 Tissue Core Kit (Macherey-Nagel) following the manufacturer’s protocol. For each variant constitutive DNA was used as a reference. When available, the constitutive DNA was extracted from the patient the lymphocytes of the corresponding patient.

Patient medical records were reviewed in order to access clinical and pathological variables, such as age at onset of cancer, tumor (SBR) grade, histologic subtype, staging, ER, PR, and Her2 status of the tumors.

### Variant selection

Twenty-six distinct *BRCA1* germline variants included ten pathogenic, eight VUS, and eight likely benign/benign variants (Supplementary Data 1). The criteria for classification were based on the French variant database from the UNICANCER Genetic Group (UGG, Unicancer)^1,17^. The eight VUS have been reported in ClinVar, but with low or medium review status. To date, they have a discordant ClinVar clinical significance (VUS, likely pathogenic, and pathogenic) and remain unclassified based on multifactorial analysis^18^.

### Pyrosequencing

Pyrosequencing was the method applied to detect allelic imbalance of the variant for the majority of tumor samples. This method quantifies the level of the nucleotide at a designated variant locus. The DNA of a patient not carrying the variant in question was used as an internal control. The analysis was performed in triplicate. A mixture (10 μl of PCR product, 7 μl of Streptavidin Sepharose beads, 25 μl of nuclease-free water, 40 μl of PyroMark binding buffer) was agitated during 10 min at 1650 rpm to bind PCR products to the beads. The beads were then captured using the vacuum workstation, washed in 40 ml of 70% ethanol for 3 s, denatured by denaturation buffer for 3 s, and then washed in wash buffer for 5 s. The beads were then released and the purified DNA samples were annealed to the sequencing primer in 25 μl of annealing buffer for 2 min at 85 °C and cooled at room temperature for 10 min. Pyrosequencing was then performed according to manufacturer protocol on a QiagenPyromark Q24 system. Pyrograms were manually interpreted using the Pyromark Q24 software. DNA of a patient known not carrying the variant in question was used as an internal control. The analysis was performed in triplicate. The patient’s tumor result was compared with her correspondent germline result when the later was available. The allelic imbalance was considered once the mutant/wild-type imbalance was superior to 10%.

### Next-generation sequencing

For six tumors, LOH analysis was performed only by amplicon-based NGS in the Ion Proton platform. LOH was considered when the variant⁄WT allele imbalance was above 10%. Primers for the c.4963T>C; p.(Ser1655Pro) *BRCA1* variant were designed using Primer3 software^40^. Libraries were prepared using Ion Plus Fragment Library Kit (Thermo Fisher Scientific) after PCR amplification. Sequencing was performed in the Ion Proton platform (Thermo Fisher Scientific), according to the manufacturer’s instructions. Mapping of sequencing reads and variant calling were performed using the Torrent Suite Browser and TVC (Thermo Fisher Scientific). Reference/variant bases coverage and frequency were inspected and annotated manually, using the Integrative Genomics Viewer (IGV) software^41^.

### Statistical analyses

Each variant had a triplicate estimation on the allelic frequency. The comparison of the average allelic frequency and the standard deviation related to the repetition of the test was based on *t* student test.

We used the chi-squared test to calculate the probability of samples with PV being (statistically significant) enriched for loss of WT allele when compared to samples of BV, as well as the probability of observing the increased loss of WT allele according to the effect of the variant at the protein level.

Next, we designed a simulation study to estimate the minimum number of cases and the number of LOH cases which would allow the classification of the variant (Supplementary Tables 2 and 3). In our model, the variable “WT LOH” follows the binomial distribution with parameters n ∈ ${\mathbb{N}}$ and p ∈ [0,1] X ~ B(n, p). The probability of getting exactly k WT LOH in n independent samples is given by the equation (1):

$$f\left( {k,n,p} \right) = \Pr \left( {k,n,p} \right) = \Pr \left( {X = k} \right) = \left( {\begin{array}{*{20}{c}} n \\ k \end{array}} \right)p^k(1 - p)^{n - k}$$

$$\left( {\begin{array}{*{20}{c}} n \\ k \end{array}} \right) = \frac{{n!}}{{k!\left( {n - k} \right)!}}$$

The first scenario was based on the probability for a PV to present a LOH if multiple cases were assessed. The second scenario was based on the probability for a BV to present a LOH. We mimicked the number of cases and the number of minimum LOH cases to classify the variant. The threshold was determined to have at least 90% probability to reach the number of LOH with PV and less than 5% probability to reach the number of LOH with BV. More details are provided in supplementary Tables 2–3 and 6–8.

### *BRCA1* promoter hypermethylation analysis

First, EpiTectBissulfite Kit (Qiagen) was used for bisulfite conversion of the tumor DNA. Next, pyrosequencing using PyroMark Q96 evaluated the methylation status of four *BRCA1* promoter CpG sites, according to the manufacturer’s protocol^42^.

### PI3K mutation analysis

The PI3K-AKT-mTOR pathway plays a crucial role in breast tumorigenesis. The presence of a mutation in this pathway could indirectly suggest that cancer development was not directly related to *BRCA1* PV. Aiming to put in evidence an alternative mechanism of carcinogenesis for tumors of pathogenic mutation presenting allelic balance, we performed a mutation screening of PIK3CA exons 9 and 20 by high-resolution melting (HRM) followed by Sanger sequencing for confirmation if a mutation was found. For HRM analysis 10 ng of DNA was amplified in a final volume of 10 μl. PCR reactions were performed using LightCycler 480 High-Resolution Melting Master.

### BRCAness signature

Lastly, when frozen samples were available (*n* = 12), the presence of the homologous recombination deficiency was assessed. The BRCAness signature was developed on the large state transition (HRD-LST) scores from CytoScan data –— signature LST—by Popova et al.^9^.

### Purification of *BRCA1* BRCT domain variants

The BRCTs variants cloned into the pETM-30 expression vector were introduced into the E. coli BL21(DE3) strain and were grown at 37°C in LB medium containing 75 mg/mL kanamycin to an OD600 nm of 1.0. Protein overproduction was induced at 20 °C with 1 mmol/L isopropyl b-d-thiogalactoside (IPTG) for 12 h. The bacteria were then harvested by low-speed centrifugation at 6000 rpm for 15 min. The bacterial pellet was suspended in 30 mL lysis buffer [100 mmol/L Tris pH 7.5, 150 mmol/L NaCl, 1% (w/v) Triton X-100, 10% glycerol, 1 mmol/L EDTA, 10 mmol/L DTT, 1 mmol/L phenylmethylsulfonyl fluoride (PMSF), 2 mmol/L ATP, 10 mmol/L MgSO4] and incubated with 1% lysozyme overnight at 4 °C. Then, 1 mL benzonase (Sigma), 2 mmol/L ATP, 10 mmol/L MgSO4, 10 mmol/L MgCl2, and 10 mmol/L DTT were added. After 30 min, the extract was centrifuged at 20,000 rpm for 30 min, and the soluble protein fraction was loaded on a 30 mL Glutathione Sepharose 4FF column previously equilibrated in 50 mmol/L Tris, 150 mmol/L NaCl pH 7.5. The column was washed with a buffer containing 1 mol/L NaCl, then 150 mmol/L NaCl, and then with 50 mmol/L Tris, 150 mmol/L NaCl pH 7.5. The TEV protease was added and incubated overnight at 4 °C. After TEV cleavage, the elution from the GST-trap column was loaded onto a HisTrap column equilibrated in 50 mmol/L TrispH7.5, 150 mmol/L NaCl. The protein was eluted with 50 mmol/L Tris pH 7.5, 150 mmol/L NaCl, and 10 mmol/L imidazoles. Fractions highly enriched in BRCT variants were diluted fivefold in 50 mmol/L Tris pH 7.5 and loaded on a 5 mL Q-Sepharose High-performance column equilibrated in 50 mmol/L Tris pH 7.5. Bound proteins were eluted with between 200 and 350 mmol/L NaCl. In both cases, the collected fractions were analyzed by 0.1% SDS-15% PAGE, using as a marker the broad range pre-stained protein marker (Bio-Rad).

### HR assays in human RG37-shBRCA1 cells

Experiments were carried out in RG37-shBRCA1 cell lines. RG37 cells are SV40-transformed human fibroblasts containing a chromosomally integrated DR-GFP substrate that specifically monitors gene conversion. RG37 cells were infected with lentiviral particles containing an shRNA directed against the 3′ UTR part of the BRCA1 messenger and the puromycin resistance gene. Puromycin was added 3 days after infection and cells were reseeded at low density to isolate individual clones. Clones were then screened for deficiency in the formation of BRCA1 foci after ionizing radiation, deficiency in HR (using the DR-GFP substrate), and for the extinction of BRCA1 expression monitored by Western blot analysis. The new cell line was called RG37-shBRCA1.

Cells were pretreated (or not) with 10 mg/mL of doxycycline 3 days before plating. They were then seeded at 2 × 10^5^ per well in six-well plates. Twenty-four hours after plating, expression vectors encoding for BRCA1 (or its variant forms) and the HA-tagged meganuclease I-SceI were cotransfected using JetPEI reagent (PolyPlus, Ozyme). Forty-eight hours after transfection, cells were trypsinized and GFP + cells were directly measured by flow cytometry.

### Multifactorial likelihood analysis and calculation of posterior probability

For each variant, the likelihood ratio derived from co-segregation analysis was combined with other sources of data to generate an overall likelihood score of causality. The probability that the variant is causal has been calculated from co-segregation data using a Bayesian statistical model described by Thompson^43^. These likelihood ratios estimates were subsequently refined by using the multifactorial model elaborated by D. Goldgar, which takes into account various data sources including data on family history and tumor characteristics^44^.

Information on segregation was recorded for all families, and likelihood ratios (LRs) based on tumor pathology, co-occurrence, and family history were available for two variants: (Table 7). The multifactorial analysis incorporates likehoods for segregation, tumor pathology, co-occurrence, and family history^18,19,22,39,45–48^. Family History and co-occurrence LRs were derived by querying an ENIGMA consortium dataset of 243 *BRCA1* and *BRCA2* tests^49^.

### Reporting Summary

Further information on research design is available in the Nature Research Reporting Summary linked to this article.

## Supplementary information

Supplementary Information

Reporting Summary

Supplementary Data 1

## Data Availability

All sequence variants have been submitted to UMD-BRCA1 (http://www.umd.be/), now called FrOG (French OncoGenetics) database, and to ClinVar database All material and data supporting this study are described within the manuscript (or by request to the authors if this is the case). Accession codes for the ClinVar data (June-2021): RCV000675184.4, RCV001357954.1, and RCV000414437.1. All data generated or analyzed during this study are included in this published article (and its supplementary information files).
