# Supplementary material for: Value of the loss of heterozygosity to BRCA1 variant classification
Source: NPJ Breast Cancer. 2022 Jan 17;8:9. doi: 10.1038/s41523-021-00361-2 (PMC8764043; doi:10.1038/s41523-021-00361-2)
Supplement: Supplementary file 1 — Supplementary Information [file 41523_2021_361_MOESM1_ESM.pdf]

Supplementary data

Supplementary Tables

Supplementary Table 2: Binomial distribution of the probability of the wild-type loss of heterozygosity (WT-LOH) for a benign variant. Grey cells delimitate the zone determined by simulation study where the probability WT-LOH for a benign variant is less than 5% and in bold the situations in which the probability is lower than 0,05%. For example, the likelihood that a benign variant will present 5 out of 5 samples with LOH is below 0,05%.

	Number of LOH sample									
Number of tested samples	1	2	3	4	5	6	7	8	9	10
1	22,22%	-	-	-	-	-	-	-	-	-
2	39,51%	4,94%	-	-	-	-	-	-	-	-
3	52,95%	12,62%	1,10%	-	-	-	-	-	-	-
4	63,40%	21,58%	3,66%	0,24%	-	-	-	-	-	-
5	71,54%	30,88%	7,64%	1,00%	**0,05%**	-	-	-	-	-
6	77,86%	39,91%	12,80%	2,48%	0,26%	**0,01%**	-	-	-	-
7	82,78%	48,35%	18,83%	4,77%	0,76%	0,07%	**0,00%**	-	-	-
8	86,61%	56,00%	25,39%	7,90%	1,65%	0,22%	**0,02%**	**0,00%**	-	-
9	89,58%	62,80%	32,19%	11,78%	3,04%	0,54%	0,06%	**0,00%**	**0,00%**	-
10	91,90%	68,75%	38,99%	16,32%	4,98%	1,09%	0,17%	**0,02%**	**0,00%**	**0,00%**
11	93,70%	73,90%	45,61%	21,36%	7,50%	1,96%	0,37%	**0,05%**	**0,00%**	**0,00%**
12	95,10%	78,30%	51,89%	26,75%	10,58%	3,19%	0,73%	0,12%	**0,01%**	**0,00%**
13	96,19%	82,03%	57,76%	32,33%	14,17%	4,83%	1,27%	0,26%	**0,04%**	**0,00%**
14	97,04%	85,18%	63,15%	37,98%	18,21%	6,91%	2,06%	0,48%	0,09%	**0,01%**
15	97,69%	87,81%	68,05%	43,58%	22,60%	9,42%	3,14%	0,83%	0,18%	**0,03%**
16	98,21%	90,01%	72,44%	49,01%	27,26%	12,35%	4,54%	1,35%	0,32%	0,06%
17	98,61%	91,83%	76,34%	54,22%	32,10%	15,66%	6,27%	2,06%	0,55%	0,12%
18	98,92%	93,34%	79,78%	59,14%	37,01%	19,31%	8,36%	2,99%	0,88%	0,21%
19	99,16%	94,58%	82,80%	63,73%	41,93%	23,25%	10,79%	4,18%	1,35%	0,36%
20	99,34%	95,59%	85,41%	67,96%	46,77%	27,40%	13,56%	5,65%	1,98%	0,58%

Supplementary Table 3: Binomial distribution of the probability of WT-LOH for a pathogenic variant. Grey cells delimitate the zone determined by simulation study where the probability of WT-LOH for a pathogenic variant is more than 90%. For example, the likelihood that a pathogenic variant will present 5 out of 5 samples with LOH is 13%. With 10 samples, the probability for a causal variant to have 5 LOH is upper 90% and while for a neutral variant it is under 5% (Supp Table S2).

Number of tested samples \ Number of LOH sample	1	2	3	4	5	6	7	8	9	10
1	65,45%	-	-	-	-	-	-	-	-	-
2	88,07%	42,84%	-	-	-	-	-	-	-	-
3	95,88%	72,44%	28,04%	-	-	-	-	-	-	-
4	98,58%	87,78%	57,11%	18,36%	-	-	-	-	-	-
5	99,51%	94,85%	77,18%	43,72%	12,01%	-	-	-	-	-
6	99,83%	97,90%	88,75%	65,62%	32,77%	7,86%	-	-	-	-
7	99,94%	99,16%	94,74%	80,76%	54,27%	24,16%	5,15%	-	-	-
8	99,98%	99,67%	97,63%	89,91%	71,61%	43,87%	17,59%	3,37%	-	-
9	99,99%	99,87%	98,97%	94,96%	83,59%	62,03%	34,79%	12,68%	2,21%	-
10	100,00%	99,95%	99,56%	97,58%	91,03%	76,14%	52,62%	27,15%	9,06%	1,44%
11	100,00%	99,98%	99,82%	98,88%	95,32%	85,89%	68,01%	43,82%	20,90%	6,43%
12	100,00%	99,99%	99,92%	99,49%	97,65%	92,06%	79,71%	59,66%	35,91%	15,90%
13	100,00%	100,00%	99,97%	99,78%	98,86%	95,72%	87,80%	72,78%	51,45%	29,00%
14	100,00%	100,00%	99,99%	99,90%	99,46%	97,77%	92,98%	82,61%	65,41%	43,69%
15	100,00%	100,00%	100,00%	99,96%	99,75%	98,88%	96,12%	89,40%	76,67%	57,91%
16	100,00%	100,00%	100,00%	99,98%	99,89%	99,45%	97,92%	93,80%	85,00%	70,19%
17	100,00%	100,00%	100,00%	99,99%	99,95%	99,73%	98,92%	96,50%	90,76%	79,89%
18	100,00%	100,00%	100,00%	100,00%	99,98%	99,87%	99,45%	98,08%	94,51%	87,00%
19	100,00%	100,00%	100,00%	100,00%	99,99%	99,94%	99,73%	98,98%	96,85%	91,92%
20	100,00%	100,00%	100,00%	100,00%	100,00%	99,97%	99,87%	99,47%	98,24%	95,15%

Supplementary Table 4 Alternative second allele inactivation mechanisms searched for pathogenic variants of breast cancer samples presenting allelic balance in pyrosequencing analysis. Of note, NGS was able to identify 5 additional cases with allelic imbalance.

**Variant**	**Co-occurence *BRCA1/2* germline pathogenic variant**	**Promoter methylation**	**LOH NGS**	**Cellularity**	**Additional variant with tumoral *BRCA1/2* sequencing**	**BRCAness analysis**	***PIK3CA* mutation**	**TNBC**	**Conclusion**
c.68_69del	No	No	No	40%	No	Low	No	Unknown	Allelic balance
c.68_69del	Unknown	No	-	75%	-	-	No	Yes	Allelic balance
dupEx3-8	No	No	-	Unknown	-	-	-	Yes	Allelic balance
c.131G>T	Unknown	No	No	Unknown	No	-	No	Yes	Allelic balance
c.131G>T	Unknown	No	-	Unknown	-	-	No	Yes	Allelic balance
c.181T>G	No	No	No	Unknown	-	No	**Yes**	No	Allelic balance
c.181T>G	Unknown	No	No	Unknown	-	-	**Yes**	Unknown	Allelic balance
c.181T>G	Unknown	No	No	Unknown	-	-	**Yes**	Unknown	Allelic balance/
c.181T>G	Unknown	No	-	Unknown	-	-	**Yes**	Unknown	Allelic balance
c.181T>G	Unknown	No	-	Unknown	-	-	**No**	Unknown	Allelic balance
c.5123C>A	Unknown	**Yes**	-	Unknown	-	-	No	Yes	Loss of variant allele/Pr methylation
c.5266dup	Unknown	No	No	50%	No	-	No	Yes	Allelic balance
c.5095C>T	No	No	-	60%	-	-	NE	Unknown	Allelic balance
c.5266dup	Unknown	NE	NE	30%	NE	NE	No	No	Allelic balance
c.5266dup	No	No	-	Unknown	No	-	No	Yes	Loss of variant allele
c.5266dup	Unknown	No	NE	Unknown	NE	-	NE	Unknown	Allelic balance
c.5453A>G	No	No	No	70%	No	Low	**Yes**	No	Allelic balance/

Supplementary Table 5: Correlation between the presence of locus specific LOH evaluated by pyrosequencing or NGS and the BRCAness score. This score uses single-nucleotide polymorphism arrays to define a signature of BRCA1-associated genomic instability. P= Pathogenic, B= Benign, VUS=Variant of Uncertain Significance.

Variant nomenclature	Protein nomenclature	Variant Classification	Type of tumor	Conclusion LOH (Pyrosequencing /NGS)	BRCAness	LOH BRCA1 (SNP array)	LOH BRCA2 (SNP array)
c.68_69del	p.Glu23Valfs*17	P	Breast	Allelic balance	Low	No	No
c.5324T>G	p.Met1775Arg	P	Breast	Loss of wt	High	Yes	Yes
c.5324T>G	p.Met1775Arg	P	Ovary	Loss of wt	High	Yes	Yes
c.5453A>G	p.(Gly1803Glnfs*11)	P	Breast	Allelic balance	Low	No	No
c.962G>A	p.Trp321*	P	Breast	Loss of wt	High	Yes	Yes
c.962G>A	p.Trp321*	P	Breast	Loss of wt	High	Yes	Yes
c.4956G>A	p.Met1652Thr	B	Breast	Allelic balance	Low	No	No
c.4956G>A	p.Met1652Thr	B	Breast	Allelic balance	Low	No	No
c.4956G>A	p.Met1652Thr	B	Breast	Allelic balance	Low	No	No
c.3074C>T	p.Thr1025Ile	VUS	Breast	Allelic balance	Low	Yes	No
c.4841C>T	p.Pro1614Leu	VUS	Breast	Loss of variant allele	Low	Yes	No
c.4841C>T	p.Pro1614Leu	VUS	Breast	Loss of wt	Low	No	No

Supplementary Table 6 Proposed application of LOH information for BRCA1 variant classification. “I”, insufficient probability to exclude neutrality, “M” between 0,05% and 5% trend in favor to exclude neutrality and “S” possibility to exclude the neutrality below 0,05%. In this context, the situation “S” lead more to a classification toward class 4. The situation “M” and “S” can be used for the classification.

Number of tested samples \ Number of LOH sample	1	2	3	4	5	6	7	8	9	10
1	I	-	-	-	-	-	-	-	-	-
2	I	M	-	-	-	-	-	-	-	-
3	I	I	M	-	-	-	-	-	-	-
4	I	I	M	M	-	-	-	-	-	-
5	I	I	I	M	S	-	-	-	-	-
6	I	I	I	M	M	S	-	-	-	-
7	I	I	I	M	M	M	S	-	-	-
8	I	I	I	I	M	M	S	S	-	-
9	I	I	I	I	M	M	M	S	S	-
10	I	I	I	I	M	M	M	S	S	S
11	I	I	I	I	I	M	M	M	S	S
12	I	I	I	I	I	M	M	M	S	S
13	I	I	I	I	I	M	M	M	S	S
14	I	I	I	I	I	I	M	M	M	S
15	I	I	I	I	I	I	M	M	M	S
16	I	I	I	I	I	I	M	M	M	M
17	I	I	I	I	I	I	I	M	M	M
18	I	I	I	I	I	I	I	M	M	M
19	I	I	I	I	I	I	I	M	M	M
20	I	I	I	I	I	I	I	I	M	M

Supplementary Table 7 Proposed application of LOH information for BRCA1 variant classification. In fact, if there is few LOH, the probability to have a PV is reduced. With “I”, insufficient probability to exclude pathogenity, “M” between 0,05% and 5% trend in favor to exclude pathogenicity, and “S” possibility to exclude the pathogenity, below 0,05%. The situation “M” and “S” can be used for the classification.

Number of tested samples	Number of LOH sample: 1	2	3	4	5	6	7	8	9	10
1	I	-	-	-	-	-	-	-	-	-
2	I	I	-	-	-	-	-	-	-	-
3	M	I	I	-	-	-	-	-	-	-
4	M	I	I	I	-	-	-	-	-	-
5	M	M	I	I	I	-	-	-	-	-
6	M	M	I	I	I	I	-	-	-	-
7	S	M	M	I	I	I	I	-	-	-
8	S	M	M	I	I	I	I	I	-	-
9	S	M	M	M	I	I	I	I	I	-
10	S	S	M	M	I	I	I	I	I	I
11	S	S	M	M	M	I	I	I	I	I
12	S	S	M	M	M	I	I	I	I	I
13	S	S	S	M	M	M	I	I	I	I
14	S	S	S	M	M	M	I	I	I	I
15	S	S	S	S	M	M	M	I	I	I
16	S	S	S	S	M	M	M	M	I	I
17	S	S	S	S	S	M	M	M	I	I
18	S	S	S	S	S	M	M	M	M	I
19	S	S	S	S	S	S	M	M	M	I
20	S	S	S	S	S	S	M	M	M	M

Supplementary Table 8: Unilateral estimation of the power. Estimate of the number of cases for BRCA1 variant classification using the power prop test.. With the probability of LOH in BV and PV, to conclude with a first risk at 5 to 10% and a power of 80%, the number of samples analyzed for variant classification should be between 10 and 15 with an unilateral evaluation.

n Sample	α = 0,01	α = 0,05	α = 0,10
1	2,91%	12,88%	23,40%
2	5,47%	20,07%	33,26%
3	8,44%	26,95%	41,76%
4	11,77%	33,55%	49,26%
5	15,39%	39,83%	55,90%
6	19,25%	45,74%	61,76%
7	23,27%	51,27%	66,93%
8	27,39%	56,40%	71,47%
9	31,58%	61,12%	75,44%
10	35,76%	65,43%	78,91%
11	39,91%	69,36%	81,92%
12	43,98%	72,92%	84,53%
13	47,95%	76,13%	86,79%
14	51,79%	79,01%	88,74%
15	55,48%	81,58%	90,42%
16	59,00%	83,88%	91,86%
17	62,34%	85,91%	93,09%
18	65,51%	87,72%	94,15%
19	68,48%	89,31%	95,05%
20	71,27%	90,71%	95,81%
21	73,87%	91,94%	96,47%
22	76,29%	93,02%	97,02%
23	78,53%	93,97%	97,49%
24	80,60%	94,79%	97,89%
25	82,50%	95,51%	98,23%
26	84,25%	96,13%	98,51%
27	85,84%	96,68%	98,75%
28	87,30%	97,15%	98,95%
29	88,63%	97,55%	99,12%
30	89,83%	97,90%	99,27%

Supplementary Figures

Supplementary Figure 1

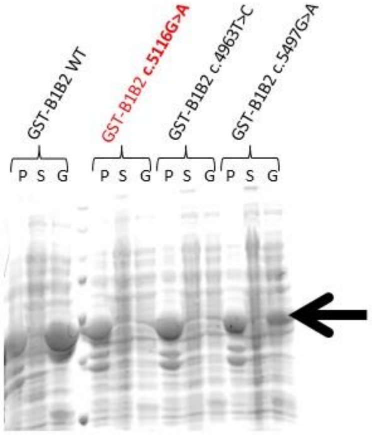

Supp Figure 1: **Classification of the VUS as a function of the impact of the corresponding missense variations on BRCT domain expression in *E. coli*.** Mutated BRCT domains fused to GST were expressed in *E. coli* and purified by affinity chromatography using glutathione beads. This figure shows a SDS-PAGE gel with samples from the bacterial pellet (P), supernatant (S) and the glutathione beads (G) after incubation with the supernatant and washing. The arrow indicates the position of the protein GST-B1B2.

Supplementary Figure 2A

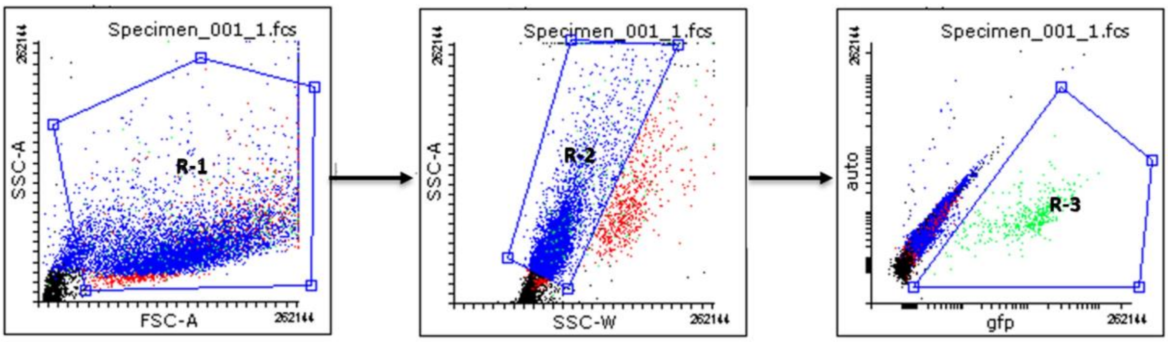

R1 84,1% Cell (not cellular debris)

R2 92,54% no cell doublets

R3 4,5% GFP cells

Supp Figure 2A: **Sorting Strategy**. Gating scheme for fluorescent-activated cell sorting of GFP-positive human fibroblasts cells RG37(R3). Cellular debris and Cell Doublets were excluded (R1 and R2).

Supplementary Figure 2B

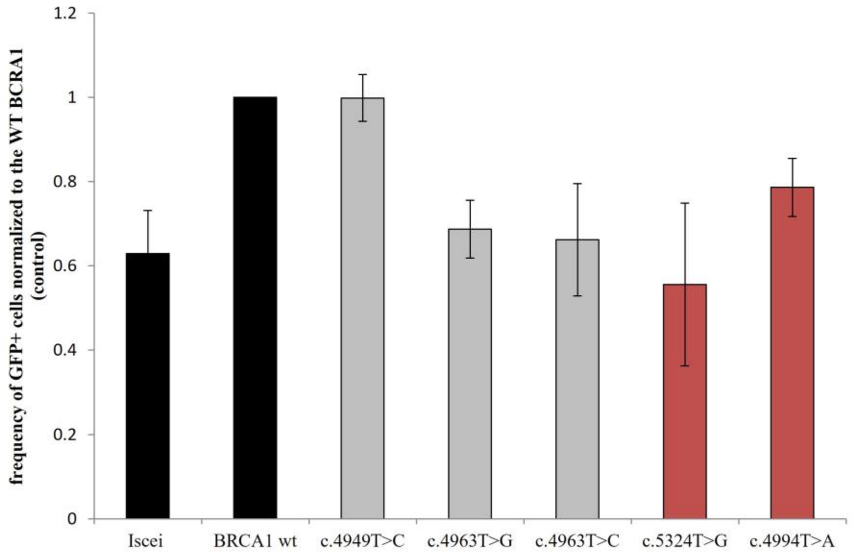

Supp Figure 2**: Impact of the BRCA1 missense mutations on HR**. Plot of the HR efficiencies measured after expression of either WT BRCA1 or VUS normalized to the HR efficiency after expression of WT BRCA1. The values are shown normalized to WT-BRCA1 (in black). Positive controls with known pathogenic variants are in red. They correspond to four to four independent experiments.

Supplementary Figure 3

	LR pathology >1	LR pathology <1
Loss of wt using LOH	30	3
Loss of mt allele/ allelic balance using LOH	13	5
Total	43	8

Pathogenic variants

	LR pathology >1	LR pathology <1
Loss of wt using LOH	2	2
Loss of mt allele/ allelic balance using LOH	2	19
Total	4	21

Benign variants

Supp Figure 3: Comparison of LOH status with LR pathology score. Consistency was observed in both analysis for most samples of pathogenic (30/51 samples presenting LR pathology>1 and Loss of wt allele) and benign variants (16/25 samples presenting LR pathology<1 and allelic balance/Loss of mt allele). One sample carrying a benign variant was not included in this analysis because LR pathology could not be calculated since it corresponded to in situ breast carcinoma.

Supplementary Figure 4

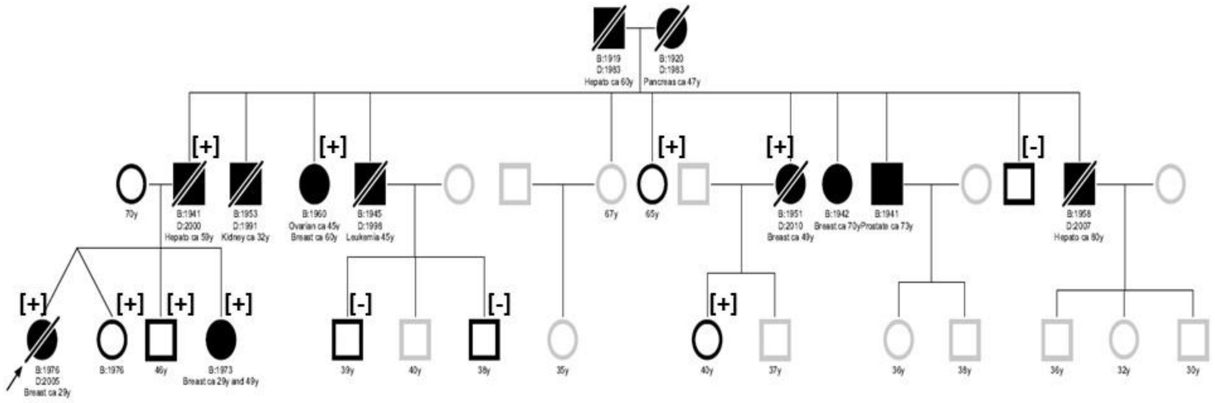

Supp Figure 4: Pedigree of the family carrying the c.4963T>C variant, showing co-segregation of the variant with breast and ovarian cancers. ca= cancer; [+]: carrier of variant; [-]: non-carrier of variant

Supplementary Figure 5

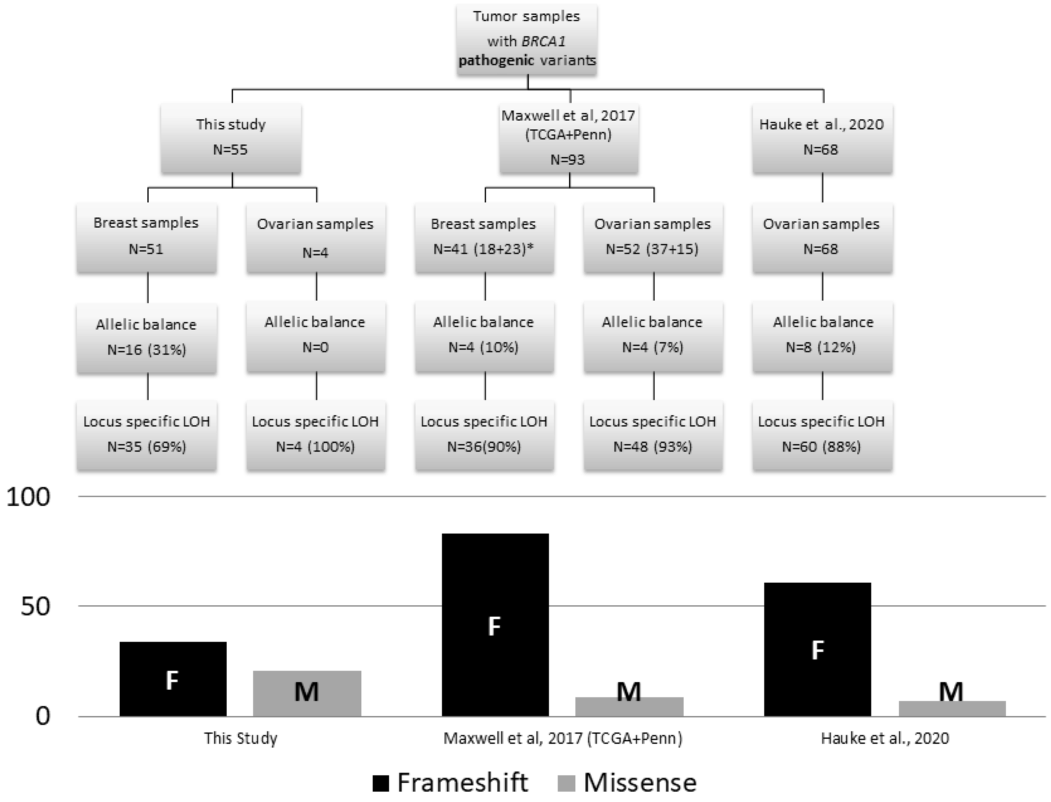

Supplementary Figure 5: Comparison between previous and current results of *BRCA* locus specific LOH status of breast and ovarian tumors from individuals with germline *BRCA1* **pathogenic** variants.

kConFab Investigators

Adrienne Sexton[18], Alexa Kidd[19], Alice Christian[19], Alison Colley[20], Alison Trainer[21], Amanda Spurdle[6], Amy Pearn[22], Andrew Fellows[23], Andrew Shelling[24], Anna deFazio[25], Ashley Crook[26], Bettina Meiser[27], Bridget Robinson[28], Briony Patterson[29], Cass Hoskins[30], Christobel Saunders[31], Clara Gaff[32], Clare Hunt[33], Clare Scott[34], David Amor[35], David Gallego Ortega[36], Deborah Marsh[37], Deepa Chauhan[38], Edwina Rickard[39], Elizabeth Salisbury[40], Ellen Pieper[41], Gelareh Farshid[42], Geoff Lindeman[43], Georgia Chenevix-Trench[44], Georgina Fenton[45], Graham Mann[46], Heather Thorne[6], Ian Bennett[47], Ian Campbell[6], Ingrid Winship[48], James Cui[49], James Flanagan[50], James Kollias[51], Jane Visvader[52], Jessica Taylor[18], Jessica Koehler[27], Jo Burke[53], Joanne Dixon[54], Jodi Saunus[55], John Hopper[56], Jonathan Beesley[57], Judy Kirk[39], Juliet French[58], Kathy Tucker[27], Kelly Phillips[59], Lara Lipton[60], Laura Forrest[61], Leon Botes[27], Lesley Andrews[27], Liz Caldon[62], Liz Lobb[63], Logan Walker[64], Manisha Chauhan[65], Margaret Cummings[66], Marion Harris[56], Mark Jenkins[30], Martin Delatycki[67], Mary Ann Young[68], Meagan Brennan[69], Melissa Brown[70], Melissa Southey[71], Michael Bogwitz[18], Michael Buckley[72], Michael Field[73], Michael Friedlander[74], Mike Gattas[75], Mitchell Lawrence[76], Mona Saleh[77], Nick Hayward[44], Nick Pachter[18], Paul Cohen[78], Paul James[33], Peter Simpson[44], Peter Fong[79], Peter George[80], Phyllis Butow[81], Rachael Williams[65], Rebecca Dickson[26], Renea Taylor[82], Rodney Scott[83], Roger Milne[84], Rosemary Balleine[85], Sarah O'Sullivan[86], Sarah-Jane Dawson[87], Shona O'Connell[88], Shuai Li[89], Sian Greening[90], Sophine Nightingale[59], Stacey Edwards[70], Stephen Fox[23], Stewart Hart[91], Sue Anne McLachlan[92], Sunil Lakhani[44], Ted Edkins[93], Yoland Antill[59]

[18] Familial Cancer Centre, Royal Melbourne Hospital, Victoria, Australia
[19]Clinical Genetics Departments, Central Regional Genetics Service,New Zealand
[20]Department of Clinical Genetics ,Liverpool Health Service ,Liverpool, Australia
[21]University of NSW, Prince of Wales Hospital, Randwick, Australia
[22]The Gene Council, Perth, Australia
[23]Molecular Diagnostic Development, Pathology Department, Peter MacCallum Cancer Centre, Melbourne , Australia
[24]Obstetrics and Gynaecology, University of Auckland, New Zealand
[25]Dept. Gynaecological Oncology, Westmead Institute for Cancer Research, Westmead, Sidney, Australia
[26] Department of Clinical Genetics, Royal North Shore Hospital, St Leonards, Australia
[27] Hereditary Cancer Clinic, Prince of Wales Hospital , Randwick, Australia
NSW 2031
[28] Oncology Service,Christchurch Hospita,Christchurch, New Zealand
[29] Tas Clinical Genetics Service, Royal Hobart Hospital, Hobart Tasmania, Australia
epartment of Clinical Oncology, A.C. Camargo Cancer Center, São Paulo, Brazil.
[30] Parkville Familial Cancer Centre, Peter MacCallum Cancer Centre & The Royal Melbourne Hospital, Melbourne, Australia
[31] School of Surgery and Pathology, QE11 Medical Centre, Nedlands, Australia
[32]Victorian Clinical Genetics Service, Royal Melbourne Hospital, Melbourne, Australia
[33] Monash Medical Centre, Victoria, Australia
[34] Research Department, Royal Melbourne Hospital , Victoria, Australia
[35] Genetic Health Services Victoria, Royal Children's Hospital, Melbourne, Australia

[36] Tumour Development Group, Garvan Institute of Medical Research,The Kinghorn Cancer Centre, Australia
[37] Kolling Institute of Medical Research, Royal North Shore Hospital, St Leonards , Australia
[38] School of Psychology, University of Sydney, Sidney, Australia
[39] Familial Cancer center, Westmead Hospital, Westmead, Australia
[40] Anatomical Pathology , Prince of Wales Hospital, Randwick, Australia
[41] Parkville Familial Cancer Centre and Genomic Medicine, Melbourne,Australia
[42] Tissue Pathology, IMVS, Adelaide, Australia
[43] Breast Cancer Laboratory, Walter and Eliza Hall Institute PO Royal Melbourne Hospital, Victoria, Australia
[44] Queensland Institute of Medical Research , Royal Brisbane Hospital, Australia
[45] South West Family Cancer Clinic, Liverpool Hospital, Liverpool, Australia
[46] Westmead Institute for Cancer Research, Westmead, Australia
[47] Silverton Place, Brisbane, Australia
[48] Department of Genetics, Royal Melbourne Hospital, Victoria, Australia
[49] Epidemiology and Preventive Medicine, Monash University, Prahan, Victoria, Australia
[50] Epigenetics Unit, Department of Surgery and Oncology, Imperial College London, London, England
[51] Breast Endocrine and Surgical Unit, Royal Adelaide Hospital, Australia
[52] The Walter and Eliza Hall Institute of Medical Research, Royal Melbourne Hospital, Victoria, Australia
[53] ICON Cancer Care, Hobart, Australia
[54] Central Regional Genetic Services, Wellington Hospital, Wellington, New Zealand
[55] Breast Pathology, University of Queensland Centre for Clinical Research, Royal Brisbane and Women's Hospital, Australia
[56] Centre for M.E.G.A., University of Melbourne, Victoria, Australia
[57] Research Officer, Queensland Institute of Medical Research, Herston, Australia
[58] School of Molecular and Microbial Sciences, University of Queensland, Australia
[59] Department of Medical Oncology, Peter MacCallum Cancer Centre, Australia
[60] Medical Oncology and Clinical Hematology Unit, Western Hospital, Australia
[61]Psychosocial Cancer Genetics Research Group, Parkville Familial Cancer Centre, Melbourne, Australia
[62]Replication and Genome Stability Cancer Division, Garvan Institute of Medical Research, Sydney, Australia
[63] Medical Psychology Research Unit, The University of Sydney, Sydney, Australia
[64]Molecular Cancer Epidemiology Laboratory, Queensland Institute of Medical Research, P.O. Royal Brisbane Hospital, Australia
[65]St Vincents Hospital Cancer Genetics Clinic, The Kinghorn Cancer Centre, Sydney, Australia
[66]Department of Pathology, University of Queensland Medical School, Australia
[67]Clinical Genetics Austin Health, Heidelberg Repatriation Hospital, Australia
[68]Genome.One, Darlinghurst, Australia
[69]NSW Breast Cancer Institute, Westmead, Australia
[70]Department of Biochemistry, University of Queensland, Australia
[71]Genetic Epidemiology Laboratory, Departemnt of Pathology, University of Melbourne, Australia
[72]Molecular and Cytogenetics Unit, Prince of Wales Hospital, Australia
[73]Royal North Shore Hospital, St Leonards, Australia

[74]Department of Medical Oncology, Prince of Wales Hospital, Australia
[75]Queensland Clinical Genetic Service, Royal Children's Hospital, Australia
[76]Prostate Cancer Research Program, Monash University, Australia
[77]Centre for Genetic Education, Prince of Wales Hospital, Australia
[78]Director of Gynaecological Cancer Research, St John of God Subiaco Hospital, Australia
[79]Medical Oncology Department, Auckland City, New Zealand
[80]Clinical Biochemistry Unit, Canterbury Health Labs, New Zealand
[81]Medical Psychology Unit , Royal Prince Alfred Hospital
[82]Cancer Program. Monash University, Australia
[83] Pathology Service, John Hunter Hospital, Australia
[84]Centro Nacional de Investigaciones Oncologicas, Madrid, Spain
[85]Westmead Institute for Medical Research,Sydney, Australia
[86]Genetic Services of Western, King Edward Memorial Hospital, Australia
[87]Molecular Genetics Department, Cambridge University,England
[88]Southern Health Familial Cancer Centre, Victoria, Australia
[89]Centre for Epidemiology and Biostatistics, Melbourne School of Population and Global Health, The University of Melbourne, Australia
[90]Illawarra Cancer Centre,Wollongong Hospital, Australia
[91]Breast and Ovarian Cancer Genetics, Monash Medical Centre Australia
[92]Department of Oncology, St Vincent's Hospital, Australia
[93]Clinical Chemistry, Princess Margret Hospital for Children, Australia
